# Identification and functional characterization of a rice NAC gene involved in the regulation of leaf senescence

**DOI:** 10.1186/1471-2229-13-132

**Published:** 2013-09-12

**Authors:** Yong Zhou, Weifeng Huang, Li Liu, Taiyu Chen, Fei Zhou, Yongjun Lin

**Affiliations:** 1 National Key Laboratory of Crop Genetic Improvement and National Centre of Plant Gene Research, Huazhong Agricultural University, Wuhan, 430070, China; 2 Plant Reproductive Biology, Mail Stop 5, University of California, 1 Shields Avenue, Davis, CA 95616-8780, USA

**Keywords:** Chlorophyll, Leaf senescence, NAC, JA, Rice (*Oryza sativa* L.)

## Abstract

**Background:**

As the final stage of leaf development, leaf senescence may cause the decline of photosynthesis and gradual reduction of carbon assimilation, which makes it a possible limiting factor for crop yield. NACs are plant-specific transcription factors and some NACs have been confirmed to play important roles in regulating leaf senescence.

**Results:**

In this study, we reported a member of the NAC transcription factor family named *OsNAP* whose expression is associated with leaf senescence, and investigated its preliminary function during the process of leaf senescence. The results of qRT-PCR showed that the *OsNAP* transcripts were accumulated gradually in response to leaf senescence and treatment with methyl jasmonic acid (MeJA). A subcellular localization assay indicated that OsNAP is a nuclear-localized protein. Yeast one-hybrid experiments indicated that OsNAP can bind the NAC recognition site (NACRS)-like sequence. *OsNAP*-overexpressing transgenic plants displayed an accelerated leaf senescence phenotype at the grain-filling stage, which might be caused by the elevated JA levels and the increased expression of the JA biosynthesis-related genes *LOX2* and *AOC1*, and showed enhanced tolerance ability to MeJA treatment at the seedling stage. Nevertheless, the leaf senescence process was delayed in *OsNAP* RNAi transgenic plants with a dramatic drop in JA levels and with decreased expression levels of the JA biosynthesis-related genes *AOS2*, *AOC1* and *OPR7*.

**Conclusions:**

These results suggest that OsNAP acts as a positive regulator of leaf senescence and this regulation may occur via the JA pathway.

## Background

Leaf senescence is complex in that it involves many highly organized molecular and cellular processes such as the disintegration of chloroplast, down-regulation of photosynthesis, degradation of nucleic acid, protein, and lipid and recycling of nutrients. And as the final stage of leaf development, leaf senescence eventually leads to leaf death, which is controlled by both internal and external factors. The internal factors include age, phytohormone levels and developmental processes, and the external factors mainly comprise environmental/biological stresses such as extreme temperature, shading, drought, wounding, nutrient limitation, pathogen attack and oxidative stress by UV-B irradiation and ozone [1-3].

NACs (NAM, ATAF and CUC) are plant-specific transcription factors and are widely found in plants. It has been reported that many NACs show enhanced expression during dark-induced and natural leaf senescence in Arabidopsis [4,5], and may play a central role in mediating leaf senescence. The senescence-controlling NAC gene *NAM-B1* was also reported to be associated with the contents of grain protein, zinc, and iron in wheat [6]. *ANAC092/AtNAC2/ORE1*, whose expression correlates with senescence, positively regulates aging-induced cell death in Arabidopsis leaves [7]. The *oresara1* (*ore1*) mutant, which lacks the functional *ANAC092*/*AtNAC2*/*ORE1* gene, displays a delayed leaf senescence phenotype [8]. An ABA-responsive NAC transcription factor VND-INTERACTING2 (VNI2), whose expression shows a leaf aging or leaf longevity-dependent expression pattern, may mediate the crosstalk between the salt stress response and the leaf aging process [9]. *AtNAP* is strongly up-regulated during leaf senescence in Arabidopsis, and *atnap* null mutants show a delayed leaf senescence phenotype, whereas the inducible overexpression of *AtNAP* causes precocious leaf senescence [10]. Recent studies have shown that AtNAP can bind to the promoter region of *SENESCENCE-ASSOCIATED GENE113* (*SAG113*) to form a ABA-AtNAP-SAG113 protein phosphastase 2C regulatory chain for controlling stomatal movement and water loss in senescing Arabidopsis leaves [11,12]. Many NAC transcription factors can be induced by leaf senescence, but their particular roles in leaf senescence still remain largely unknown.

Many studies have revealed that JA and its derivatives play important roles in regulating the response to leaf senescence in plants. MeJA and its precursor JA were first isolated in oat and shown to promote senescence in detached oat leaves, suggesting that jasmonates might serve as powerful promoters to induce plant senescence [13]. JA-induced leaf senescence is accompanied by the increased expression of several enzymes involved in JA biosynthesis and the decreased expression of the genes involved in photosynthesis [14-16]. In addition, JA can activate the expression of many senescence-regulated genes such as *AtWRKY6*[17], *OsAkaGal*[18] and *ESR/ESP*[19], which play major roles in leaf senescence. Leaf senescence and MeJA can induce the expression of *OsAkaGal*, which encodes a chloroplast alkaline α-galactosidase involved in the degradation of digalactosyl diacylglycerol during leaf senescence in rice [18,20]. Exogenous application of JA to attached and detached leaves promotes leaf senescence in Arabidopsis but does not induce leaf senescence in the JA-insensitive mutant *coi1*, suggesting that the JA-signaling pathway is required for JA to promote leaf senescence [14]. Furthermore, JA promotes H_2_O_2_ accumulation in the leaves of JA-sensitive cultivar TN1 seedlings to accelerate the process of senescence but not in the leaves of JA-insensitive cultivar TNG67 [21]. Many JA and senescence-regulated genes have been identified, yet how the crosstalk between JA signaling and senescence occurs remains to be thoroughly understood.

In this study, we isolated and characterized the JA-induced senescence-associated gene *OsNAP*, which encodes a NAC transcription factor in rice. We generated the *OsNAP* overexpression and RNAi lines and analyzed the leaf senescence process of them. We also determined the endogenous JA levels and checked the expression of the genes encoding the enzymes of the JA biosynthetic pathway in the *OsNAP* transgenic lines and wild-type plants. Studies of these lines indicated that OsNAP acts as a positive regulator of the JA pathway to mediate the leaf senescence process in rice.

## Results

### Characterization of *OsNAP* in rice

There are 75 predicted NAC proteins [22] and 140 putative NAC or NAC-like proteins (ONAC) in rice [23]. Later computational analyses showed that there are at least 151 OsNAC genes in the rice genome [24]. And phylogenetic analysis showed that there are 13 OsNACs which closely cluster with AtNAP: Os03g21060 (OsNAP), Os12g03040, Os11g03300 (OsNAC10), Os03g60080 (SNAC1/OsNAC9), Os01g60020 (OsNAC4), Os07g12340 (OsNAC3), Os11g08210 (OsNAC5), Os07g37920, Os05g34310, Os07g48450, Os01g01430, Os05g34830 and Os01g66120 (OsNAC6/SNAC2) (Additional file 1: Figure S1). Digital expression profile analysis showed that the expression levels of Os07g37920, Os05g34310, Os07g48450 and Os01g01430 are low and even undetectable in leaves. And other 9 genes are expressed in leaves, but only the transcripts of *OsNAP*, *OsNAC5* and *OsNAC6/SNAC2* in leaves are up-regulated during leaf senescence [25-27]. However, several studies have demonstrated that OsNAC5 and OsNAC6/SNAC2 are involved in stress tolerance in rice [28-30]. We therefore decided to examine the potential role of OsNAP in the leaf senescence of rice.

The full-length cDNA of *OsNAP* was isolated for further functional analysis. *OsNAP* cDNA (Accession number AK243514) encodes a protein with 392 amino acids. Sequence analysis suggested that *OsNAP* is identical to *ONAC058*, which belongs to the NAC family in rice [23]. Twelve sequences were obtained from *Arabidopsis thaliana*, *Oryza sativa*, *Populus trichocarpa*, *Gossypium hirsutum*, *Glycine max*, *Bambusa emeiensis*, *Brachypodium distachyon*, *Sorghum bicolor*, *Hordeum vulgare*, and *Zea mays* by BLASTP search. The resulting phylogenetic relationships showed that each of them contained A-E subdomains (Additional file 1: Figure S2), which is consistent with the results previously reported [31,32].

### Subcellular localization of OsNAP

**Figure 1:**
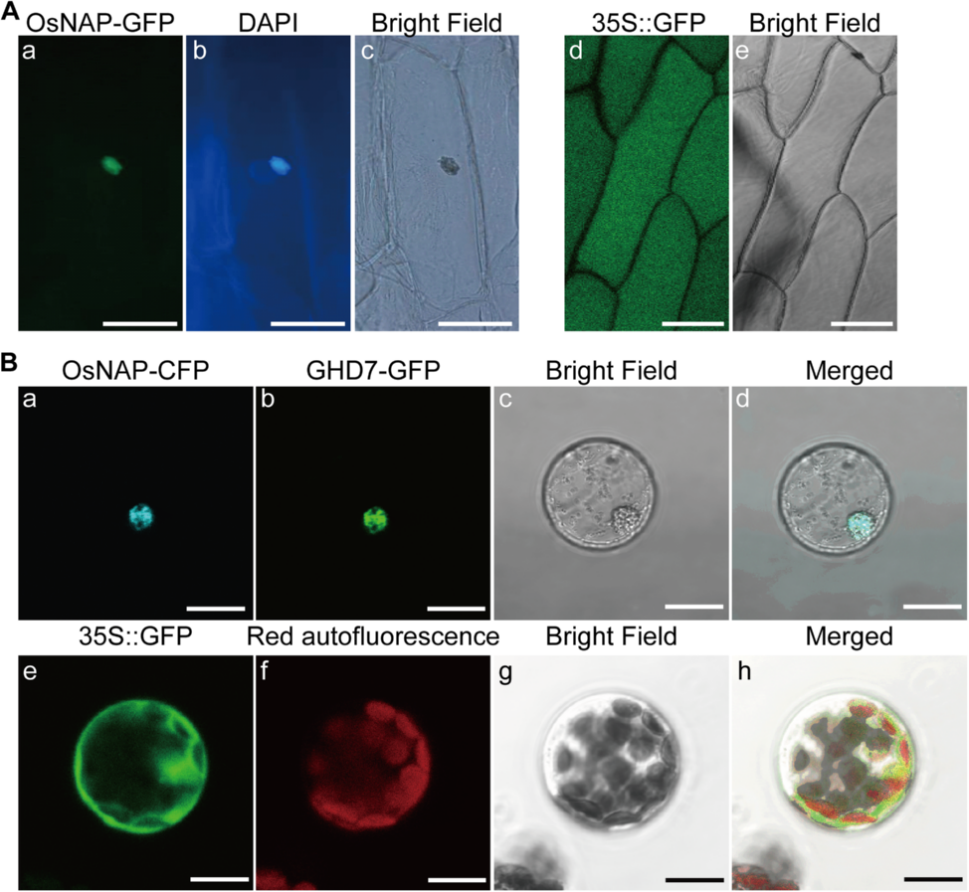
**Subcellular localization of OsNAP. A**, Nuclear localization of OsNAP in onion cells. OsNAP cDNA was fused to GFP under control of the maize (*Zea mays*) ubiquitin promoter and the construct was expressed in onion cells. (a) The transfected cells expressing GFP were photographed; (b) DAPI stained; (c) Bright field. (d) and (e), GFP alone. The bar indicates 80 μm. **B**, Nuclear localization of OsNAP in Arabidopsis protoplast. Arabidopsis cell protoplasts were transformed using 35S::*OsNAP*-GFP and 35S::*Ghd7*-CFP. Fluorescence signals (GFP and CFP) were detected by a laser confocal-scanning microscope. (a), 35S::*Ghd7*-CFP; (b), 35S::*OsNAP*-GFP; (c), Bright field; (d), overlap images of (a), (b) and (c). (e), (f) indicated autofluorescence emitted by chloroplasts; (g), Bright field; (h), overlap images of (e), (f) and (g). The bar indicates 10 μm.

To determine the subcellular localization of OsNAP, a construct expressing *OsNAP* linked to *GFP* under the control of the maize (*Zea mays*) ubiquitin promoter was transiently transfected into onion cells. The fluorescence was observed only in the nuclei, which was confirmed by DAPI staining (Figure 1A). To further study the localization of OsNAP, Arabidopsis cell protoplasts were co-transformed using 35S::*OsNAP*-GFP and 35S::*Ghd7*-CFP. As shown in Figure 1B, GFP fluorescence from OsNAP overlapped with the CFP signals from GHD7 [33], which further confirmed that OsNAP localizes to the nucleus.

### Biochemical function of OsNAP in yeast

**Figure 2:**
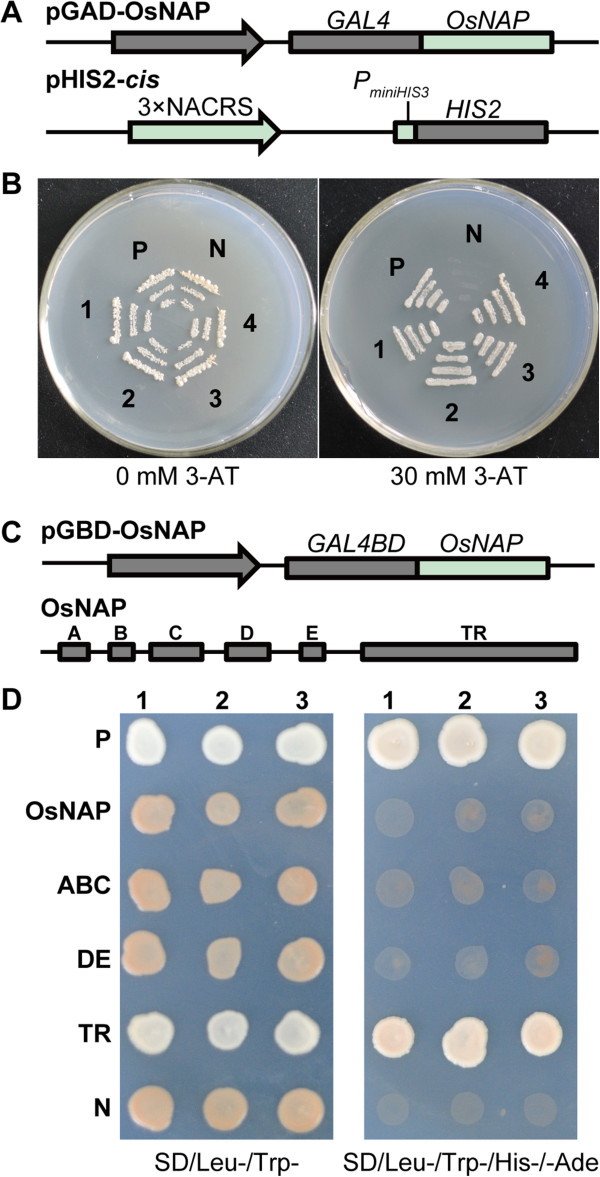
**Biochemical function of OsNAP in yeast. A**, Constructs for the yeast one-hybrid assay. **B**, OsNAP can bind to the NACRS-like sequence in yeast. The pGAD-OsNAP plasmid and reporter construct pHIS-*cis*[34] were cotransformed into the Y187 yeast strain. The transformants were examined by growth performance on SD/Leu-/Trp-/His- plates with or without 30 mM 3-AT. N, negative control (p53HIS2 plus pGAD-OsNAP); P, positive control (p53HIS plus pGAD-Rec2-53); lables 1–4, four different colonies containing pGAD-OsNAP and pHIS-*cis*. **C**, Constructs of transactivational analysis. **D**, Transactivation assay of OsNAP in yeast. The full-length and partial fragments of OsNAP were ligated into the pGBDKT7 vector and co-transformed into the AH109 yeast strain with pGADT7 to verify the transactivation activity. The transformants were examined by growth performance on SD plates in the absence or presence of histidine and the necessary amino acids. N, negative control (pGBDK7/pGADT7); P, positive control (pHIS53/pGAD-Rec2-53); lables 1–3, three different colonies containing positive control, pGBD-OsNAP/pGADT7, pGBD-OsNAP-ABC/pGADT7, pGBD-OsNAP-DE/pGADT7, pGBD-OsNAP-TR/pGADT7 and negative control, respectively.

Some NAC transcriptional factors bind to the NAC recognition site (NACRS)-like sequence in the *OsERD1* (*early responsive to drought 1*) promoter to activate *HIS* reporter gene expression [30,34-36]. To detect whether OsNAP can bind to this sequence, the ORF of *OsNAP* was fused to GAL4-AD in the pGADT7 vector and co-transformed with the pHIS2-*cis* reporter vector [34] into the Y187 yeast strain (Figure 2A). The combinations of pGAD-Rec2-53/p53HIS and pGAD-OsNAP/p53HIS2 were used as the positive control and negative control, respectively. The result showed that all of the combinations, including pGAD-OsNAP/pHIS2-*cis*, the positive and negative control, could grow on the SD/Leu-/Trp-/His- medium without 3-AT. However, when the SD/Leu-/Trp-/His- medium was supplemented with 30 mM 3-AT, the transformed pGAD-OsNAP/pHIS2-*cis* and the positive control grew, whereas the negative control did not (Figure 2B), indicating that OsNAP binds to the NACRS-like sequence in yeast.

Several NACs not only act on gene promoters but also have transactivation activity [30,34,36]. To determinate whether OsNAP has activation capacity, we tested the transactivation activity of OsNAP in yeast. The full-length and partial fragments of OsNAP were fused to the GAL4 DNA binding domain in the pGBKT7 vector and the resultant vectors were co-transformed with pGADT7 into the AH109 yeast strain (Figure 2C). The combinations of pHIS53/pGAD-Rec2-53 and pGBDK7/pGADT7 were used as the positive control and negative control, respectively. It was observed that all of the co-transformants grew well on the SD/Leu-/Trp- medium, but only the cells transformed with pHIS53/pGAD-Rec2-53 and pGBD-OsNAP-TR/pGADT7 could grow on the SD/Leu-/Trp-/His-/Ade- medium (Figure 2D), suggesting that the transactivation domain of OsNAP is located in the C-terminal transcriptional regulatory (TR) domain of the protein.

### Expression pattern of *OsNAP*

**Figure 3:**
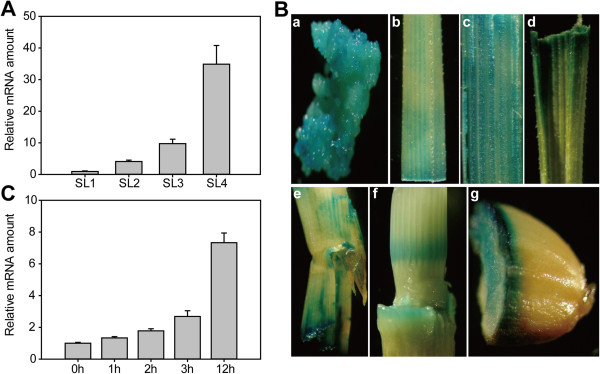
**Expression pattern of *OsNAP*. A**, qRT-PCR analysis of *OsNAP* transcript levels in flag leaves of different development periods in rice. SL1, flag leaves at the booting stage, SL2, flag leaves at the anthesis stage, SL3, flag leaves at the mature stage, SL4, flag leaves at the ripening stage. **B**, Tissue localization of OsNAP. a, callus; b, young leaf; c, mature leaf; d, senescent leaf; e, sheath; f, node and internode; g, mature seed. **C**, qRT-PCR analysis of *OsNAP* transcripts with MeJA treatment at different time points. Roots of Zhonghua 11 plants at the trefoil stage were immersed in nutrition solutions contained 0.1 mM MeJA, and several plants were collected at different points after treatment for RNA isolation.

To determine the expression profile of *OsNAP,* qRT-PCR assays using total RNA samples from flag leaves of different developmental periods were performed. The results showed that the transcript of *OsNAP* was associated with the senescence of leaves (Figure 3A). To better understand the *OsNAP* expression pattern, the *OsNAP* promoter was fused to the *GUS* reporter gene and introduced into the Zhonghua 11. Immunohistochemical staining of GUS showed that *OsNAP* was mainly expressed in callus, leaves, sheaths, nodes, internodes and mature seeds (Figure 3B). In leaves, GUS activity increased gradually with leaf senescence (Figure 3B, b-d), which is consistent with the qRT-PCR result (Figure 3A).

In addition, qRT-PCR analysis showed that the expression of *OsNAP* was continuously increased during MeJA treatment (Figure 3C), indicating that the expression of *OsNAP* was induced by MeJA treatment.

### Effect of the overexpression of *OsNAP* on leaf senescence at the grain-filling stage

**Figure 4:**
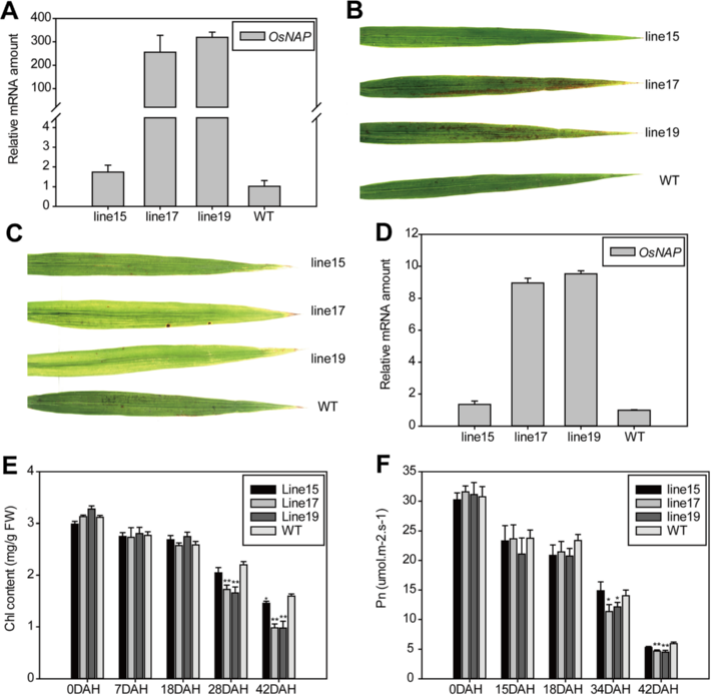
***OsNAP *overexpression lines showed an accelerated leaf senescence phenotype. A**, Expression level of *OsNAP* in the *OsNAP* overexpression lines and wild-type plants at the seedling stage. **B** and **C**, Photographs of flag leaves in *OsNAP* overexpression lines and WT plants at 30 **(B)** and 40 DAH **(C)**. **D**, Expression level of *OsNAP* in the *OsNAP* overexpression lines and wild-type plants at the grain-filling stage. The first-strand cDNAs were prepared using RNAs extracted from the flag leaves of *OsNAP* overexpression lines and WT plants at the grain-filling stage. Each value represents the mean ± SD evaluated from three replicates. **E** and **F**, Time course determinations of chlorophyll content and net photosynthetic rate in flag leaves of *OsNAP* overexpression lines and WT plants after heading. The values are means (± SD) of four replicates.

Because strongly up-regulated expression of *OsNAP* was observed during leaf senescence in rice (Figure 3A, 3B, b-d), we decided to investigate the role of *OsNAP* in leaf senescence. The *OsNAP* gene, which was driven by the constitutive cauliflower mosaic virus 35S promoter, was introduced into Zhonghua 11 by an *Agrobacterium*-mediated transformation approach. 10 single-copy insertion lines were obtained based on Southern blot analysis (Additional file 1: Figure S3). The result of the Northern blot analysis showed that the expression levels of *OsNAP* were substantially elevated in these lines (Additional file 1: Figure S4). All the plants with increased expression levels of *OsNAP* exhibited visible yellowing phenotype in leaves during the late stages of seed maturation when compared with the WT plants (data not shown). Three *OsNAP* transgenic lines (line 15, 17 and 19) were selected for further study. The expression levels of *OsNAP* in these lines were validated using qRT-PCR (Figure 4A). No significant difference was observed between the transgenic lines and WT plants during the vegetative and early reproductive stages. However, at 30 DAH, the flag leaves of the high-expressing lines (line 17 and 19) showed an accelerated yellowing phenotype when compared with those of the low-expressing line (line 15) and WT plants (Figure 4B). The yellowing in the leaves became more apparent at 40 DAH (Figure 4C). And the phenotype was correlated with the increasing expression levels of *OsNAP* (Figure 4D). The time-course changes in the chlorophyll content and net photosynthetic rate of the flag leaves were compared between the *OsNAP-*overexpressing lines and WT plants after heading. The chlorophyll content and net photosynthetic rate showed no significant differences at around 20 DAH (Figure 4E, 4F). However, the high-expressing lines (line 17 and 19) exhibited more reduction in chlorophyll content and net photosynthetic rate compared with the low-expressing line (line 15) and WT plants at around 30 and 40 DAH (Figure 4E, 4F), suggesting an accelerated leaf senescence phenotype. Taken together, these findings indicate that the overexpression of *OsNAP* leads to accelerated leaf senescence in rice.

### Effect of knock-down of *OsNAP* on leaf senescence in rice

**Figure 5:**
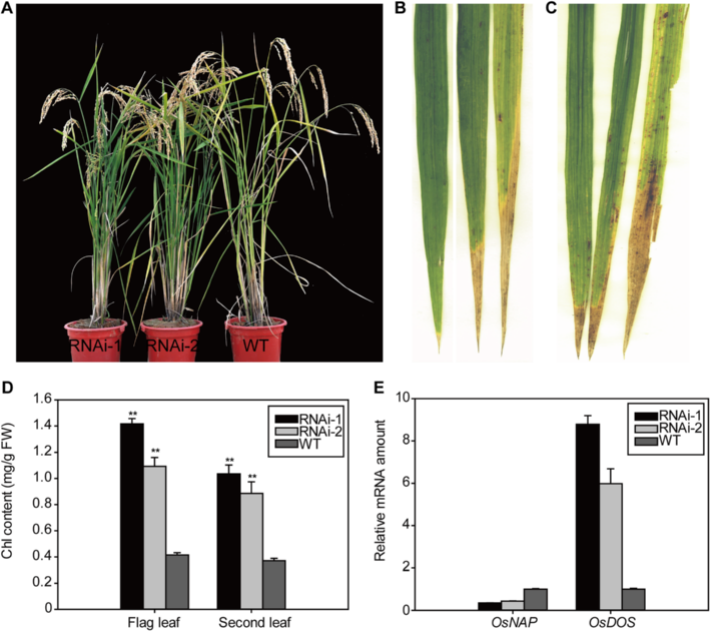
***OsNAP *RNAi transgenic plants displayed delay of leaf senescence phenotype. A**, *OsNAP* RNAi transgenic lines (RNAi-1 and RNAi-2) and WT plants at 50 DAH. **B**, flag leaves of RNAi-1, RNAi-2 and WT at 50 DAH. **C**, Second top leaves of RNAi-1, RNAi-2 and WT at 50 DAH. **D**, Content of chlorophyll of flag and second top leaves in RNAi-1, RNAi-2 and WT plants at 50 DAH. Values are means ± SD (*n* = 3). **P < 0.01 (*t* test). **E**, qRT-PCR analysis of the expression levels of *OsNAP* and *OsDOS* in flag leaves of RNAi-1, RNAi-2 and WT plants at 50 DAH. Each value represents the mean ± SD evaluated from three replicates.

To explore the physiological role of *OsNAP* in leaf senescence, we generated an *OsNAP* RNAi construct and introduced it into Zhonghua 11. 10 single-copy insertion lines were verified by Southern blot analysis (Additional file 1: Figure S5). Northern blot analysis showed that the *OsNAP* transcripts in senescing leaves decreased in most of the transgenic plants (Additional file 1: Figure S6). The leaves of RNAi transgenic lines appeared normal during the vegetative and early reproductive stages, but displayed appreciably higher greenness compared with that of WT plants during the late stages of seed maturation. Two transgenic lines (RNAi-1 and RNAi-2) were selected for further analysis. During the grain-filling stage, an obvious yellowing was observed in the leaves of the WT plants but not in those of the transgenic lines RNAi-1 and RNAi-2 (Figure 5A-C), suggesting that the senescence process was markedly delayed. We further checked the chlorophyll levels in the flag and second top leaves. The chlorophyll levels in the flag and second top leaves of RNAi-1 and RNAi-2 were sharply higher than in those of the WT plants (Figure 5D). qRT-PCR analysis validated that RNAi-1 and RNAi-2 transgenic lines showed a significant reduction in *OsNAP* transcript levels (Figure 5E). And the greater the reduction in *OsNAP* transcript levels was, the higher the chlorophyll content became in the leaves of RNAi transgenic lines compared with in those of the WT plants (Figure 5D, 5E), which indicated that the delayed leaf senescence phenotype was related to the reduction of the expression level of *OsNAP*. OsDOS acts as a negative regulator of leaf senescence since its expression is down-regulated during leaf senescence [37], which makes *OsDOS* an ideal molecular marker for leaf senescence in rice. We further analyzed the transcript of *OsDOS* by qRT-PCR analysis. As shown in Figure 5E, the expression of *OsDOS* was markedly higher in the leaves of RNAi-1 and RNAi-2 than in those of the WT plants, which further suggested that leaf senescence was remarkably delayed in *OsNAP* RNAi transgenic plants.

### Relation between the senescence phenotype in *OsNAP* transgenic plants and endogenous JA content

**Figure 6:**
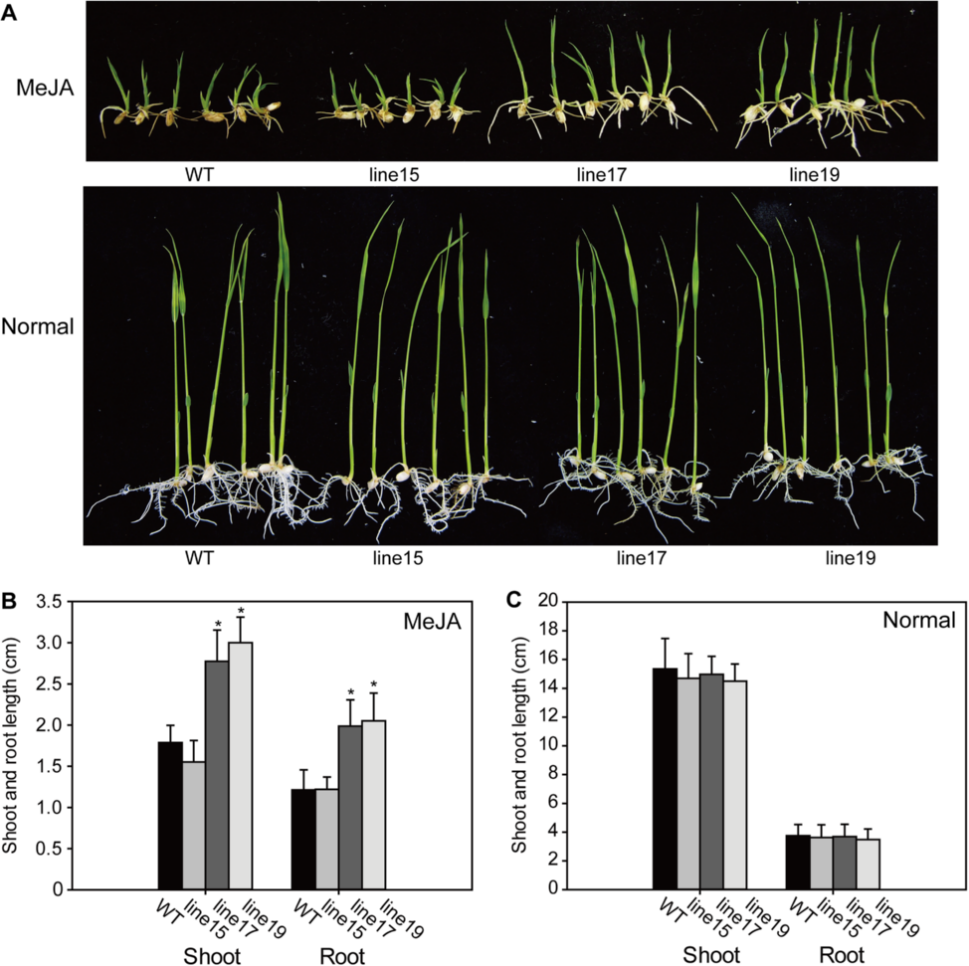
***OsNAP*-overexpressing transgenic lines showed enhanced tolerance to MeJA treatment. A**, Seeds of WT and *OsNAP*-overexpressing plants were germinated at 22°C on 1/2 MS medium and then transferred to 1/2 MS medium with and without 25 μM MeJA. The photograph was taken on day 10 after inoculation. The experiment was repeated three times. **B** and **C**, Shoot and root lengths of the lines grown on MeJA-containing **(B)** or normal **(C)** medium. The shoot and root lengths were counted after growth for 10 d. Error bars indicate SD (*n* = 10). *P < 0.05 (*t* test).

*OsNAP* expression was increased gradually by MeJA treatment (Figure 3C), implying that OsNAP may be involved in the JA pathway in rice. To assess the function of the *OsNAP* gene in the JA pathway, a MeJA treatment assay was performed on *OsNAP-*overexpressing lines and WT plants. There was no significant difference observed in the shoot and root length under normal conditions (Figure 6A). The growth of *OsNAP-*overexpressing lines and the WT plants was inhibited under MeJA conditions, but the shoot and root length of high *OsNAP*-overexpressing lines (line 17 and 19) was significantly longer than that of the low-expressing line (line 15) and WT plants (Figure 6B, 6C). However, no difference in shoot and root length was observed between the seedlings of the RNAi lines and the WT plants (data not shown). These findings indicated that the tolerance of *OsNAP*-overexpressing lines to MeJA treatment was enhanced.

**Figure 7:**
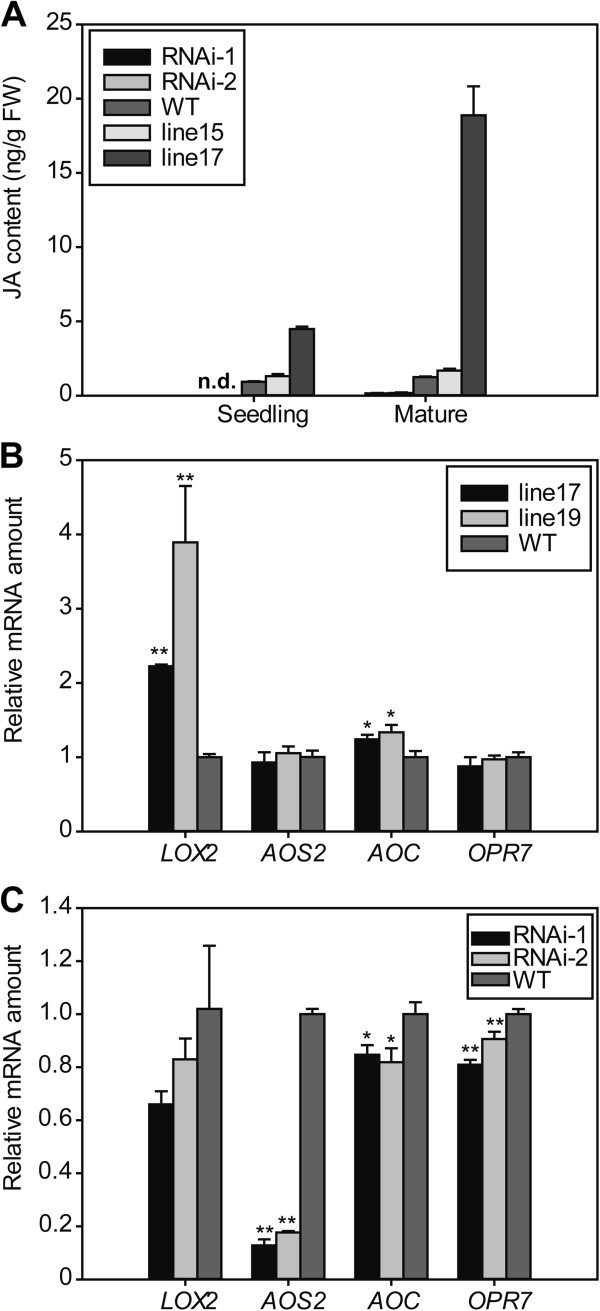
**Effects on JA content and JA biosynthetic genes in *OsNAP *transgenic lines and WT plants. A**, Endogenous levels of JA in *OsNAP* transgenic and WT plants. Leaf samples of *OsNAP*-overexpressing transgenic lines (line 15 and line 17), *OsNAP* RNAi lines (RNAi-1 and RNAi-2) and WT plants at seedling and mature stage were used for the analysis. Each point represents a mean (three replicates) ± SD. n.d., no detected. **B**, qRT-PCR analysis for JA biosynthetic genes in leaves of the *OsNAP-*overexpressing lines (line 17 and line 19) and WT plants. Transcripts associated with leaves of *OsNAP*-overexpressing lines and WT plants at the seedling stage were analyzed. **C**, qRT-PCR analysis for JA biosynthetic genes in leaves of the *OsNAP* RNAi lines and WT plants at the mature stage. The first-strand cDNAs were prepared using RNAs extracted from the leaves of *OsNAP* RNAi lines and WT plants at the mature stage. Bars represent mean (3 replicates) ± standard deviation. *P < 0.05, **P < 0.01 (*t* test).

To further explore the function of the *OsNAP* gene in the JA pathway, we measured the endogenous JA levels in *OsNAP* transgenic lines and WT plants. As shown in Figure 7A, JA levels were up-regulated in *OsNAP*-overexpressing transgenic lines, but sharply down-regulated in RNAi lines compared with those in WT plants whether at the seedling or mature stage. We further analyzed the expression levels of the genes involved in the JA biosynthetic pathway by qRT-PCR in *OsNAP* transgenic lines and the WT plants. Among these genes, *LOX2* and *AOC* had more significantly increased transcripts in the *OsNAP*-overexpressing plants than in WT plants (Figure 7B), while the genes that encode enzymes for JA biosynthesis, including *AOS2*, *AOC* and *OPR7* showed remarkably decreased expression levels in the *OsNAP* RNAi lines compared with the WT plants (Figure 7C). Therefore, the phenotypes of the *OsNAP* transgenic plants may at least be partially due to the endogenous JA levels.

## Discussion

### Important role of OsNAP in regulating leaf senescence in rice

NAC proteins are unique transcription factors involved in different developmental processes including senescence in plants [38]. Approximately one-fifth of NACs (20/109) are present in the senescence ESTs (expressed sequence tags) in Arabidopsis [5]. About 46% of the up-regulated genes in *ANAC092/AtNAC2/ORE1*-overexpressing transgenic plants are senescence-associated genes [39]. These findings suggest that NAC proteins may play crucial roles in senescence.

In this study, we identified a member of the NAC transcription factors *OsNAP*, which plays an important role in regulating leaf senescence in rice. Firstly, the transcript of *OsNAP* was up-regulated during natural leaf senescence (Figure 3A, 3B, b-d). Secondly, *OsNAP*-overexpressing lines displayed an accelerated leaf senescence phenotype and showed a reduced net photosynthetic rate coupled with a decline in chlorophyll content during the reproductive growth stage (Figure 4). In addition, *OsNAP* RNAi lines showed a markedly delayed leaf senescence, which was confirmed by the higher expression of *OsDOS* (Figure 5). Finally, as the homolog of *AtNAP* in rice, *OsNAP* can restore the delayed leaf senescence phenotype in *atnap* mutant to a normal WT phenotype [10]. These results suggest that OsNAP is associated with leaf senescence in rice.

### The role of OsNAP in the crosstalk between senescence and the JA pathway

JA and its derivatives are known as endogenous modulators of many physiological processes in plants including senescence [14,40,41]. Our analysis revealed that the expression level of *OsNAP* was up-regulated with MeJA treatment (Figure 3C), suggesting that OsNAP may be involved in the JA pathway. There are two important processes in the JA pathway: JA biosynthesis and JA signal transduction [40,42]. Accelerated or hindered JA biosynthesis can lead to more rapid or delayed senescence in plants. *OsNAP* overexpression resulted in accelerated senescence in transgenic rice and it was evident from the decreased chlorophyll content and net photosynthetic rate (Figure 4), which was correlated with accumulation of endogenous JA content and increased transcript levels of JA biosynthesis genes (Figure 7A, 7B). qRT-PCR results showed that the expression of the JA biosynthesis gene *LOX2* was increased 2 to 4 fold in the *OsNAP*-overexpressing lines compared with in the WT plants (Figure 7B). JA levels were increased during senescence in *OsNAP*-overexpression transgenic lines and the WT plants, which is in accordance with previous conclusions [2,4,15]. On one hand, JA can induce the expression of the key enzymes of chlorophyll breakdown including chlorophyllase [43], promoting the loss of chlorophyll in leaves. On the other hand, JA can also induce the expression of many senescence-associated genes to promote senescence [14,44]. Therefore, it is possible that the accelerated leaf senescence observed in *OsNAP*-overexpressing plants might have resulted from an increase in the endogenous JA levels. In addition, *OsNAP* RNAi transgenic plants displayed a marked delay of leaf senescence (Figure 5). JA in these lines declined dramatically to non-detectable levels (Figure 7A), and the transcripts of the genes encoding enzymes in the JA biosynthesis pathway, including *AOS2*, *AOC* and *OPR7,* dropped sharply (Figure 7C). The delayed yellowing during natural senescence in *OsNAP* RNAi transgenic plants at the grain-filling stage may be due to the reduced JA production. Hence, OsNAP may act as a positive regulator of leaf senescence by regulating the JA biosynthesis pathway.

Besides JA biosynthesis, a block in JA signal transduction can also alter the process of senescence. The mutant *coi1*, whose JA signaling has been impaired, exhibits delayed senescence phenotype [45]. Rubisco activase (RCA) is a COI1-dependent JA-repressed protein and it has been shown that a loss-of-function mutation of *RCA* leads to typical signs of senescence [46]. OsDOS is a nucleus-localized CCCH-type zinc finger protein in rice that acts as a negative regulator of the JA pathway and senescence process [37]. The transcripts of *OsDOS* were significantly higher in *OsNAP* RNAi lines than in the WT plants (Figure 5E), suggesting that the JA signaling pathway was impaired. In addition, MeJA inhibition was significantly lower in the *OsNAP* high-expressing lines compared with in WT plants, suggesting that the overexpression of *OsNAP* enhances the resistance to exogenous MeJA in rice (Figure 6). These results indicate that JA signaling pathway is involved in the OsNAP-mediated senescence process.

The NAC transcription factors can bind to specific *cis*-elements of target gene promoters, and can thus regulate gene transcription. For example, the miR319-regulated TCP (TEOSINTE BRANCHED/CYCLOIDEA/PCF) transcription factors regulate the JA biosynthesis gene *LOX2*, controlling JA content and affecting leaf senescence [47]. And AtNAP can bind to the promoter region of *SAG113*, which negatively regulates the ABA signaling pathway that modulates stomatal movement and water loss in senescing leaves [11]. In the present study, ABA did not have significant effects on *OsNAP* expression levels (data not shown), while MeJA gradually induced the expression of *OsNAP* (Figure 3C). In addition, OsNAP was able to bind to the NACRS-like sequence (Figure 2A, 2B), and the C-terminal region had transactivation activity in yeast (Figure 2C, 2D). It’s worth noting that there were a number of putative NAC recognition sequences (NACRS) and core DNA binding sequences (CDBS) in the 1 kb region upstream of the JA biosynthesis-related genes (Additional file 1: Table S2). For these reasons, it can be concluded that OsNAP may interact with JA biosynthesis genes directly or indirectly. Further experiments, particularly a screen for the interacting proteins, will be necessary to determine the specific roles of OsNAP in JA signaling and leaf senescence.

## Conclusions

In this article, a NAC transcription factor OsNAP was isolated and characterized in rice, and OsNAP was identified as a key mediator between the JA pathway and leaf senescence. In addition, the expression of *OsNAP* was detected in the callus, sheath, mature seed, node and internode besides the leaves (Figure 3B), suggesting that OsNAP plays important roles in diverse biologic processes in rice. Nevertheless, further analyses are required to clarify the specific roles of OsNAP in rice.

## Methods

### Plant materials and growth conditions

Zhonghua 11 (*Oryza Sativa* L*.* ssp. *Japonica* cv. Zhonghua 11) was used for this study. Wild-type and *OsNAP* transgenic plants were planted in the field of Huazhong Agricultural University (Wuhan, China).

To check the *OsNAP* expression during MeJA treatment, Zhonghua 11 plants were grown in a greenhouse with a 14-h light/10-h dark cycle. MeJA treatment was conducted by spraying 0.2 mM MeJA on the leaves. Five plants were collected at each time point of 0, 1, 2, 3 and 12 h after treatment for RNA isolation.

For MeJA treatment at the seedling stage, T_2_ seeds of Zhonghua 11 and overexpression lines were sterilized and germinated on 1/2 MS medium with 16-h light/8-h dark cycle for 4 days, and then transferred to 1/2 MS medium containing 25 μM MeJA. The shoot and root lengths of the seedlings were measured after 10 days of growth.

### Subcellular localization of OsNAP

The cDNA fragment of *OsNAP* was amplified using the full-length cDNA clone J100075D15 (http://cdna01.dna.affrc.go.jp/cDNA) as a template with the following primers: 5’- ATCggtaccATGGTTCTGTCGAACCCGGC-3’ and 5’-ATCggatccGTTCATCCCCATGTTAGAGT. The PCR products were sub-cloned into the pU1391a-GFP vector digested with *Kpn* I and *BamH* I to form pU1391a-OsNAP-GFP [48]. The construct was transformed into onion (*Allium cepa* L.) epidermal cells using a Biolistic PDS-1000/He particle delivery system (Bio-Rad) according to a previously described method [48]. After fixation in 4% PFA and staining with 10 μg/ml DAPI, transient expression of GFP fluorescence was observed using a Confocal Laser-scanning Microscope (TCS SP2, Leica, Germany).

A 1176-bp cDNA fragment of *OsNAP* was amplified using the following primers: 5’-gaattcATGGTTCTGTCGAACCCGGC-3’ and 5’-tctagaGTTCATCCCCATGTTAGAGT-3’ with a *EcoR* I site and a *Xba* I site. The PCR products were digested with *EcoR* I/*Xba* I and inserted into pM999-35S-EGFP. The OsNAP-EGFP construct and a positive control construct 35S::*Ghd7*::CFP containing a marker gene were co-introduced into Arabidopsis protoplasts as previously described [49]. The GFP and CFP fluorescences were visualized using Confocal Laser-scanning Microscopy (TCS SP2, Leica, Germany).

### Functional characterization of OsNAP in yeast

For the yeast one-hybrid assay, the cDNA fragment of *OsNAP* was amplified and cloned into the pGADT7 (Clontech, Palo Alto, CA, USA) vector to form pGAD-OsNAP. pGAD-OsNAP/pHIS2-*cis*, the positive control (pHIS53/pGAD-Rec2-53) and the negative control (pHIS2/pGAD-OsNAP) were co-transformed into the Y187 yeast strain for determination of the DNA-protein interactions according to a previous report [34]. For the transactivation assay, the PCR product of the full-length and partial fragments of *OsNAP* were fused in frame to the GAL4 DNA binding domain in the pGBDK7 vector to form pGBD-OsNAP, pGBD-OsNAP-ABC, pGBD-OsNAP-DE and pGBD-OsNAP-TR. The combinations of pGBD-OsNAP/pGADT7, pGBD-OsNAP-ABC/pGADT7, pGBD-OsNAP-DE/pGADT7, pGBD-OsNAP-TR/pGADT7, the positive control (pHIS53/pGAD-Rec2-53) and the negative control (pGBDK7/pGADT7) were co-transformed into the AH109 yeast strain for the verification of transactivation activity.

### Construction of expression plasmids and transformation into rice

To investigate the expression pattern of *OsNAP*, the promoter fragment of *OsNAP* was amplified using the following primers: 5’- ATCGaagcttCCGTTGCATTAGGAAACGTC-3’ and 5’ -ATCGtctagaACCCACACACAACACACACC-3’ and then cloned into the DX2181b vector [50], yielding the *OsNAP* promoter::GUS construct. To create the overexpression construct, the *OsNAP* genomic DNA sequence was amplified using the following primers: 5’- ATCGccatggTTCGCCATGTGCAATTATGT-3’ and 5’-ATCGggttaccCAGGGAGGTGTGTGTTGTGT-3’. The PCR fragments were digested with *Nco* I and *Bst*E II and cloned into the pCAMBIA1301 expression vector. To suppress the *OsNAP* gene, a cDNA fragment of *OsNAP* was amplified using the following primers:5’- ATCggatccCCACCACCAACAACAACAAC-3’ and 5’-ATCggtaccCTCAGTCCCAGTGACGATCC-3’ and inserted into pMCG161 and pDS1301 [51] to form pMCG161-OsNAP and pDS1301-OsNAP, respectively. The pMCG161-OsNAP plasmid was digested with *Sac* I/*Spe* I and inserted into the pDS1301-OsNAP to generate the RNAi construct. All the constructs were introduced into the EHA105 *Agrobacterium tumefaciens* strain and then introduced into the rice callus of Zhonghua 11 according to a previous method [52].

### Molecular analysis of putative transgenic plants

The putative transgenic plants were identified by PCR according to a previously described method [53]. The copy numbers of the transgenic plants were confirmed by Southern blot using a published protocol [54]. The transcripts of *OsNAP* in single-copy transgenic lines were detected by Northern blot as described previously [2]. The probes for Southern and Northern blot were amplified using PCR with gene-specific primers listed in Additional file 1: Table S1. The PCR program was as follows: 94°C for 5 min, followed by 30 cycles of 94°C for 30 s, 58°C for 30 s, and 72°C for 30 s, and finally 72°C for 7 min.

### GUS staining assay

GUS staining and observation were carried out as described previously [50]. Various tissues and organs, including the callus, leaves, sheaths, nodes and mature seeds from *P*_*OsNAP*_::*GUS* transgenic plants were immersed in GUS staining solution (0.1% Triton X-100, 1 mg/mL X-Gluc, 100 μg/ml chloramphenicol, 1 mM potassium ferricyanide, 1 mM potassium ferrocyanide, 10 mM Na_2_-EDTA, 20% methanol and 50 mM sodium phosphate, pH 7.0) at 37°C overnight. After staining, the samples were bleached with 75% (v/v) ethanol and photographed using a dissecting microscope (Leica, Germany).

### RNA isolation and quantitative RT-PCR analysis

Total RNAs were extracted using Trizol reagent according to the manufacturer’s protocol (Invitrogen, USA). First-strand cDNAs were synthesized in a reaction volume of 20 μL containing 3 μg RNase-free DNase I treated total RNA, 0.5 mg Oligo(dT)_15_, 1 mM dNTPs, 10 mM dithiothreitol, and 200 units of SuperScript™ III reverse transcriptase (Invitrogen, USA). Quantitative RT-PCR (qRT-PCR) was carried out as described previously [55]. Three replicates were performed for the analysis of each gene and for determining the relative expression levels between the repeats according to a previous report [56]. *Actin* was used as an internal control for normalization. The primers are listed in Additional file 1: Table S1.

### Measurement of chlorophyll content, net photosynthetic rate and endogenous JA levels

Chlorophyll content was determined as described previously [57]. Net photosynthetic rate was measured from 9:00 am to 11:00 am or 3:00 pm to 5:00 pm using CIRAS-2 according to the manufacturer’s instructions (PP system, USA). The endogenous JA level was extracted and quantified using the previously described method [58].

## Abbreviations

JA: Jasmonic acid; MeJA: Methyl jasmonate acid; ORF: Open reading frame; WT: Wild-type; 3-AT: 3-amino-1,2,4-triazole; TR domain: Transcriptional regulatory (TR) domain; DAH: Day after heading.

## Competing interests

The authors declare that they have no competing interests.

## Authors’ contributions

YL conceived the study and acquired the funding. LL provided the subcellular localization in onion. WH carried out the transactivation assay and JA quantification. YZ has done the other experiments and drafted the manuscript. FZ revised the manuscript. YL contributed to data interpretation and edited the final manuscript. All authors have read and approved the final manuscript.

## Supplementary Material

Additional file 1: Table S1 PCR primers used for this study. **Table S2.** Analysis of *cis*-elements in the promoter region of JA biosynthesis-related genes tested in this study. **Figure S1.** Phylogenetic analysis of AtNAP and rice NAC protein sequences. **Figure S2.** OsNAP is a NAC protein. **Figure S3.** Southern blot analysis of the *OsNAP*-overexpressing transgenic T_0_ lines. **Figure S4.** Northern blot analysis of the *OsNAP*-overexpressing transgenic lines and WT control. **Figure S5.** Southern blot analysis of the *OsNAP* RNAi T_0_ lines. **Figure S6.** Northern blot analysis of the *OsNAP* RNAi T_0_ lines and WT control. Click here for file
