# Supplementary material for: Identification and functional characterization of a rice NAC gene involved in the regulation of leaf senescence
Source: BMC Plant Biol. 2013 Sep 12;13:132. doi: 10.1186/1471-2229-13-132 (PMC3847160; doi:10.1186/1471-2229-13-132)
Supplement: Additional file 1: Table S1 — PCR primers used for this study. Table S2. Analysis of cis-elements in the promoter region of JA biosynthesis-related genes tested in this study. Figure S1. Phylogenetic analysis of AtNAP and rice NAC protein sequences. Figure S2. OsNAP is a NAC protein. Figure S3. Southern blot analysis of the OsNAP-overexpressing transgenic T0 lines. Figure S4. Northern blot analysis of the OsNAP-overexpressing transgenic lines and WT control. Figure S5. Southern blot analysis of the OsNAP RNAi T0 lines. Figure S6. Northern blot analysis of the OsNAP RNAi T0 lines and WT control. [file 1471-2229-13-132-S1.pdf]

Additional file 1, Table S1. PCR primers used for this study.

Gene	Forward primer (5'-3')	Reverse primer (5'-3')	Use
HPT	ATTTGTGTACGCCCGACAGT	GGATATGTCCTGCGGGTAAA	PCR
OsNAP	catatgATGGTTCTGTCGAACCCGGC	gaattcGTTCATCCCCATGTTAGAGT	yeast one-hybrid assay/ transactivation assay
OsNAP-ABC	catatgATGGTTCTGTCGAACCCGGC	gaattcGGTCGCCGCGCCGCCGCT	transactivation assay
OsNAP-DE	catatgAACGAGAGCGTCGGCGTC	gaattcGCTGCTCTTCTTGTAGAT	transactivation assay
OsNAP-TR	catatgCACGCGTCGCCGCTGGCC	gaattcGTTCATCCCCATGTTAGAGT	transactivation assay
OsNAP	CCACCACCAACAACAACAAC	CTCAGTCCCAGTGACGATCC	Northern blot
Actin	TGTATGCCAGTGGTCGTACCA	CCAGCAAGGTCGAGACGAA	qRT-PCR
OsNAP	AACCATTTCATCGCGAACAAC	CAGTGACGATCCCTGCAAGG	qRT-PCR
LOX2	GCATCCCCAACAGCACATC	AATAAAGATTTGGGAGTGACATATTGG	qRT-PCR
AOS2	CAATACGTGTACTGGTCGAATGG	AAGGTGTCGTACCGGAGGAA	qRT-PCR
AOC	AAGAGGAATCGAGGACAAGATATTTG	AAGCCTCTTCTTGTTCGGATCA	qRT-PCR
OPR7	GACCGCACTGACGAGTATGGT	CCACAGCCCTAGTTACCTCAAGTAG	qRT-PCR
OsDOS	ATGATGATGATGGGGGAAGG	CTCACGGGGAGGTGAGACC	qRT-PCR

Additional file 1, Table S2. Analysis of *cis*-elements in the promoter region of JA biosynthesis-related genes tested in this study.

Gene	NACRS[a]	CDBS[b]
LOX2	0	4
AOS2	1	6
AOC	1	5
OPR7	0	6

[a] Numbers of putative NAC recognition sequence (NACRS) in the 1 kb region upstream of the start codon of each gene.

[b] Numbers of putative core DNA binding sequence (CDBS) in the 1 kb region upstream of the start codon of each gene.

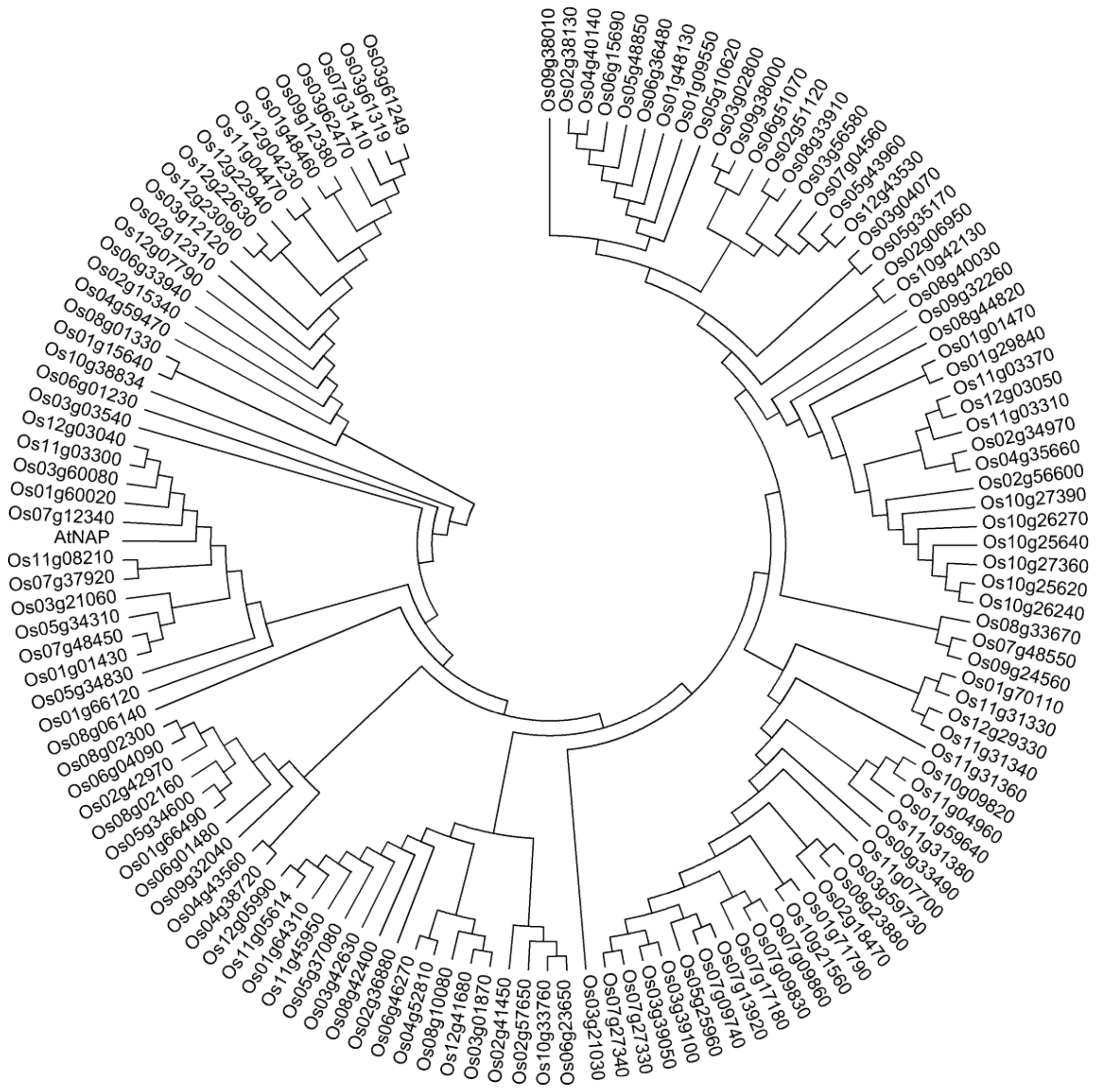

Additional file 1, Figure S1. Phylogenetic analysis of AtNAP and rice NAC protein sequences.

The locus ID of the rice NAC family genes were came from the published papers [1, 2], amino acid sequences were derived according to their locus ID from The Institute for Genomic Research (TIGR, http://www.tigr.org/). The maximum likelihood tree was constructed by the program MEGA 5.0 with default parameters [3].

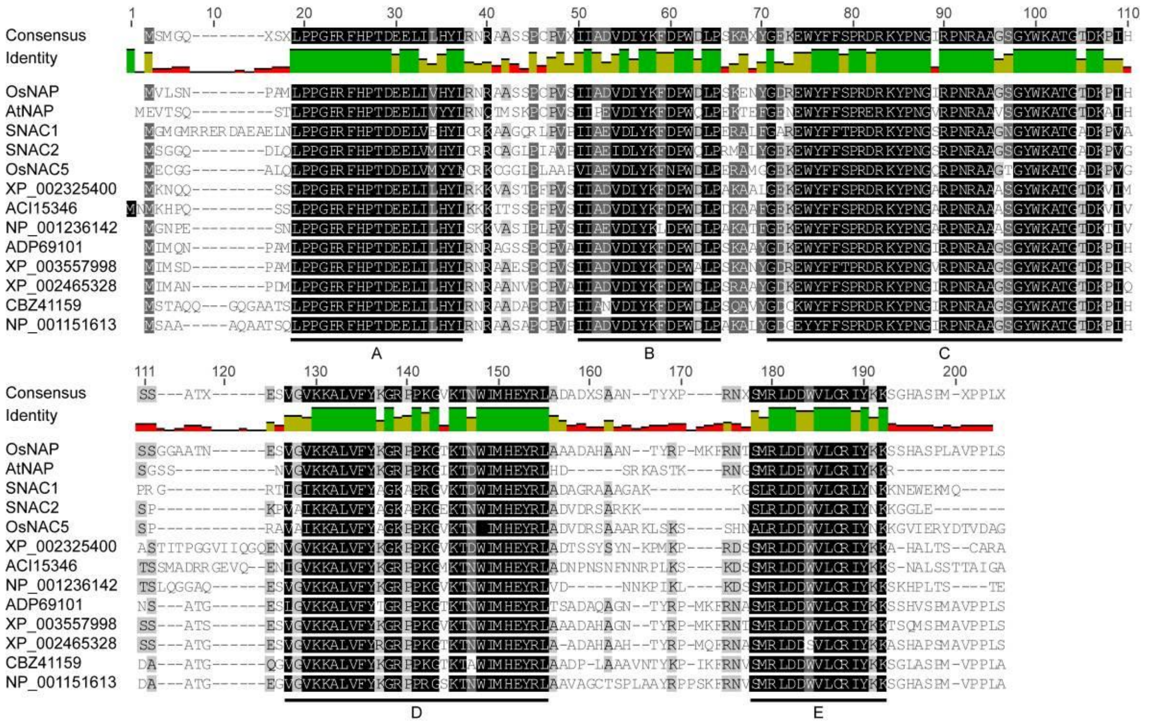

Additional file 1, Figure S2. OsNAP is a NAC protein.

The amino acid sequences of the NAC family genes from other species were aligned with OsNAP using BLAST and the multiple sequence alignments were constructed by Clustal_X with default parameters [4]. The sequence numbers are as follows: OsNAP, AAN64999, *Oryza sativa*; AtNAP, NP_564966, *Arabidopsis thaliana*; SNAC1, NP_001051682, *Oryza sativa*; SNAC2, NP_001045016, *Oryza sativa*; OsNAC5, AB028184, *Oryza sativa*; XP_002325400, *Populus trichocarpa*; ACI15346, *Gossypium hirsutum*; NP_001236142, *Glycine max*; ADP69101, *Bambusa emeiensis*; XP_003557998, *Brachypodium distachyon*; XP_002465328, *Sorghum bicolor*; CBZ41159, *Hordeum vulgare*; NP_001151613, *Zea mays*. The sections underlined and highlighted are the A to E domains in each NAC protein.

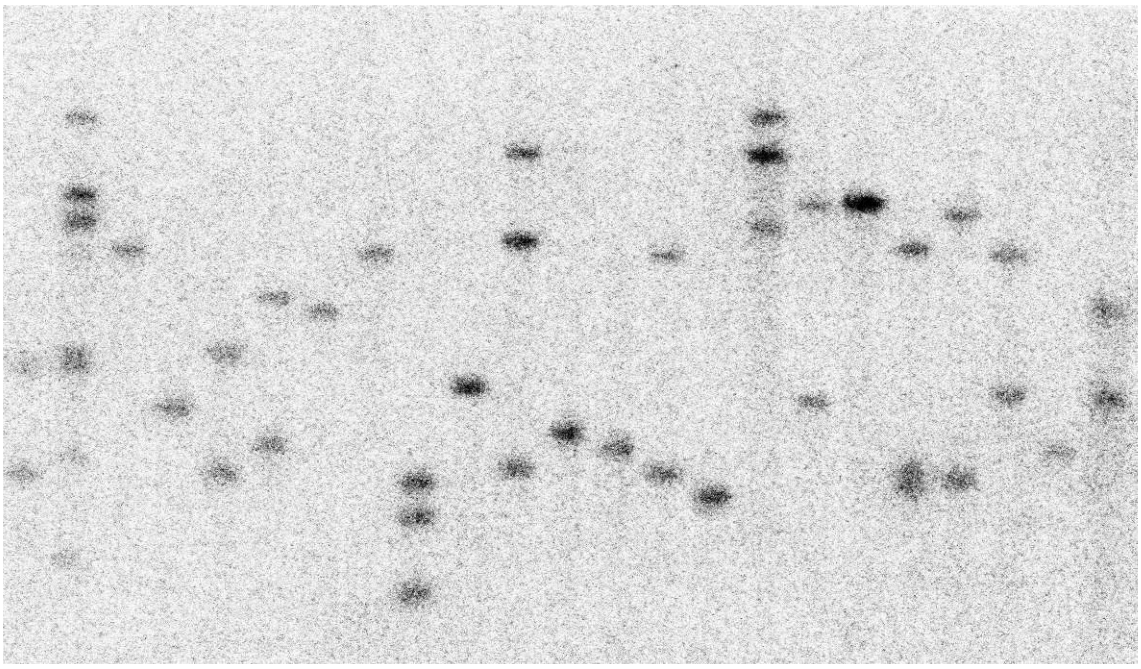

Additional file 1, Figure S3. Southern blot analysis of the *OsNAP*-overexpressing transgenic T_0 lines.

The genomic DNA of leaves was digested with *EcoR* I, transferred to Hybond nylon membrane (Amersham, USA) and hybridized with hygromycin phosphotransferase (*HPT*)-specific probe.

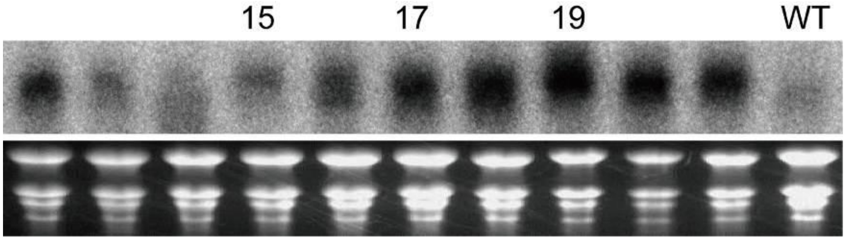

Additional file 1, Figure S4. Northern blot analysis of the *OsNAP*-overexpressing transgenic lines and WT control.

15 μg of total RNA was transferred to Hybond nylon membrane (Amersham, USA) and hybridized with *OsNAP*-specific probe. The samples were came from leaves of *OsNAP*-overexpressing single-copy T_0 lines and WT plants at the seedling stage.

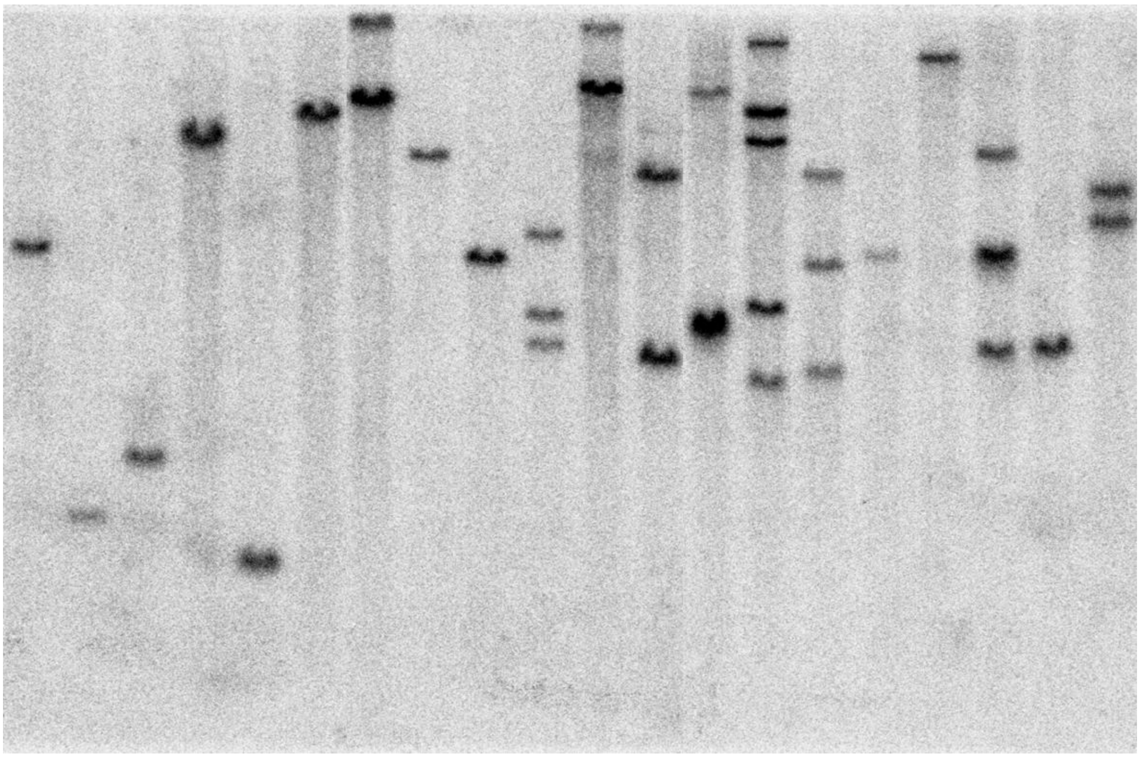

Additional file 1, Figure S5. Southern blot analysis of the *OsNAP* RNAi T_0 lines.

The genomic DNA of leaves was digested with *EcoR* I, transferred to Hybond nylon membrane (Amersham, USA) and hybridized with hygromycin phosphotransferase (*HPT*)-specific probe.

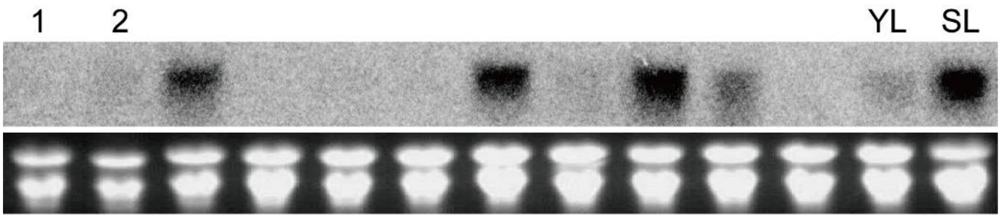

Additional file 1, Figure S6. Northern blot analysis of the *OsNAP* RNAi T_0 lines and WT control.

15 μg of total RNA was transferred to Hybond nylon membrane (Amersham, USA) and hybridized with *OsNAP*-specific probe. The RNA samples were came from flag leaves of *OsNAP* RNAi T_0 lines at the grain-filling stage. YL, young leaves of WT plants at the seedling stage; SL, flag leaves of at the grain-filling stage.

References

1. Fang Y, You J, Xie K, Xie W, Xiong L: **Systematic sequence analysis and identification of tissue-specific or stress-responsive genes of NAC transcription factor family in rice**. *Mol Genet Genomics* 2008, **280**:547-563.
2. Nuruzzaman M, Manimekalai R, Sharoni AM, Satoh K, Kondoh H, Ooka H, Kikuchi S: **Genome-wide analysis of NAC transcription factor family in rice**. *Gene* 2010, **465**:30-44.
3. Tamura K, Peterson D, Peterson N, Stecher G, Nei M, Kumar S: **MEGA5: molecular evolutionary genetics analysis using maximum likelihood, evolutionary distance, and maximum parsimony methods**. *Mol Biol Evol* 2011, **28**:2731-2739.
4. Thompson JD, Gibson TJ, Plewniak F, Jeanmougin F, Higgins DG: **The CLUSTAL_X windows interface: flexible strategies for multiple sequence alignment aided by quality analysis tools**. *Nucleic Acids Res* 1997, **25**:4876-4882.
